# Radiotherapy for Orbital Myeloid Sarcoma as Extramedullary Relapse in a Pediatric Acute Myeloid Leukemia Patient Post-haploidentical Bone Marrow Transplantation

**DOI:** 10.7759/cureus.86350

**Published:** 2025-06-19

**Authors:** Mohammadhossein Ghafouri, Ramesh Boggula, Christopher Nagle, Michael C Joiner, Steven R Miller

**Affiliations:** 1 Department of Oncology, Wayne State University School of Medicine, Detroit, USA

**Keywords:** aml treatment, general radiation oncology, orbital myeloid sarcoma, orbit metastasis, pediatric hematology-oncology

## Abstract

The proliferation of immature granulocytic cells characterizes myeloid sarcoma (MS), which can be a rare extramedullary manifestation of acute myeloid leukemia (AML). MS can occur as an isolated lesion, concurrently with AML, or as a post-treatment relapse. In this case report, we describe a 12-year-old male with high-risk AML who underwent chemotherapy followed by haploidentical hematopoietic stem cell transplantation (HSCT). Two years post-HSCT, he experienced a relapse with testicular MS, requiring orchiectomy and radiation therapy. One year later, he experienced a second relapse, with the disease isolated to the left orbit without evidence of systemic disease. Given his prior treatments, chronic graft-versus-host disease, and negative minimal residual disease, radiation therapy alone (2,400 cGy in 12 fractions) was selected to treat the left orbital MS. The dosimetric analysis showed optimal tumor coverage while sparing critical structures. He achieved a complete clinical response with preserved vision. This case highlights the complexities of managing post-HSCT MS in pediatric patients and highlights the role of targeted radiation in localized relapse when systemic therapy is not feasible.

## Introduction

Acute myeloid leukemia (AML) is a malignant disorder of the hematopoietic system characterized by the clonal proliferation of immature myeloid cells in the bone marrow, peripheral blood, or extramedullary sites [1]. AML accounts for approximately 15-20% of all childhood leukemias in pediatric patients, making it significantly less common than acute lymphoblastic leukemia (ALL), which constitutes about 75% of pediatric leukemia cases [2,3]. Despite advancements in treatment, pediatric AML remains a challenging disease due to its heterogeneity, aggressive nature, and relatively high risk of relapse compared to ALL.

Extramedullary presentation of AML, such as myeloid sarcoma (MS), is a rare phenomenon in which leukemic cells form a solid tumor outside the bone marrow. MS occurs in approximately 2-8% of AML cases and can manifest in various locations, including the skin, lymph nodes, gastrointestinal tract, and central nervous system. The orbital region is an exceptionally uncommon site for MS, making such cases noteworthy. MS may present as isolated (primary), de novo concurrently with AML, chronic myeloproliferative neoplasms (e.g., chronic myeloid leukemia), or myelodysplastic syndromes (secondary), or as a relapse after treatment, often indicating a poorer prognosis. Diagnosis can be challenging due to its ability to mimic other malignancies or inflammatory processes, and it requires histopathological confirmation, typically through biopsy [4-6].

Treatment of MS in pediatric patients with a history of AML and prior bone marrow transplantation (BMT) is multifaceted and requires a personalized approach. MS, in this context, often represents relapsed or refractory disease and requires aggressive therapy. The choice of chemotherapy depends on whether the patient is chemotherapy-naïve for relapse or has previously relapsed. Common regimens include salvage chemotherapy with FLAG-IDA (fludarabine, cytarabine, idarubicin, and granulocyte colony-stimulating factor (G-CSF)), MEC (mitoxantrone, etoposide, and cytarabine), or CLAG (cladribine, cytarabine, and G-CSF) and targeted agents (if applicable) such as FLT3 inhibitors (e.g., gilteritinib) for FLT3-mutated AML and IDH1/IDH2 inhibitors (e.g., ivosidenib or enasidenib) for IDH-mutated disease [7]. For patients unable to tolerate intensive chemotherapy, hypomethylating agents such as azacitidine or decitabine, either alone or in combination with venetoclax, have been used as treatment options [8-11].

Radiation therapy is a valuable option for patients with isolated MS who do not respond adequately to chemotherapy, those with localized recurrence following hematopoietic stem cell transplantation (HSCT), and in cases requiring rapid symptom management due to compression of critical structures, such as the spinal cord or orbit. Radiation therapy provides effective, fast-acting, and long-lasting local control at the treatment site [6,12]. Doses typically range from 1,000 to 3,000 cGy over one to three weeks, depending on the patient’s tolerance and the extent of the disease. A low-dose radiation therapy regimen of 2,400 cGy, delivered in 12 fractions, is effective for most patients, offering excellent disease control with minimal side effects. Alternatively, lower doses between 600 and 2,000 cGy, administered in 200 cGy fractions, can alleviate symptoms and reduce disease burden when a longer radiation course is impractical [13-16].

## Case presentation

Initial diagnosis and HSCT

A 12-year-old African American male was diagnosed with high-risk AML four years previously at the age of eight years. A bone marrow biopsy at that time revealed a complex karyotype, but no unfavorable cytogenetic or molecular mutations were identified. He was enrolled in the St. Jude AML16 protocol and received treatment per the decitabine arm. He subsequently underwent a haploidentical HSCT from his mother.

He experienced several early post-transplant complications, including veno-occlusive disease, febrile neutropenia, mucositis, and BK virus-associated hemorrhagic cystitis, which were resolved with appropriate management. Infectious complications included cytomegalovirus reactivation, recurrent *Clostridium difficile* infections, and infections with adenovirus and human herpesvirus-6. Chronic graft-versus-host disease (GVHD) involving the liver, musculoskeletal system, and eyes requires long-term immunosuppression, physical therapy, and other supportive methods.

First relapse: testicular MS

The patient experienced a relapse, presenting with extramedullary MS in the left testicle approximately two years after transplant. He underwent a left orchiectomy, and pathology was consistent with recurrent MS. Subsequently, postoperative radiation therapy was administered to both testes due to concerns that the right testicle could be a sanctuary site for additional disease. He received a total dose of 2,400 cGy in 12 fractions of 200 cGy per fraction to the left testicular surgical bed and the right testicle, with a 0.5 cm bolus placed over the scrotum to help increase the skin surface dose. A cradle was fashioned to stabilize the pelvis, and the penis was secured superiorly and placed out of the radiation field. The doses to the rectum and bladder were within the dose constraints for these organs.

Second relapse: orbital MS

**Figure 1:**
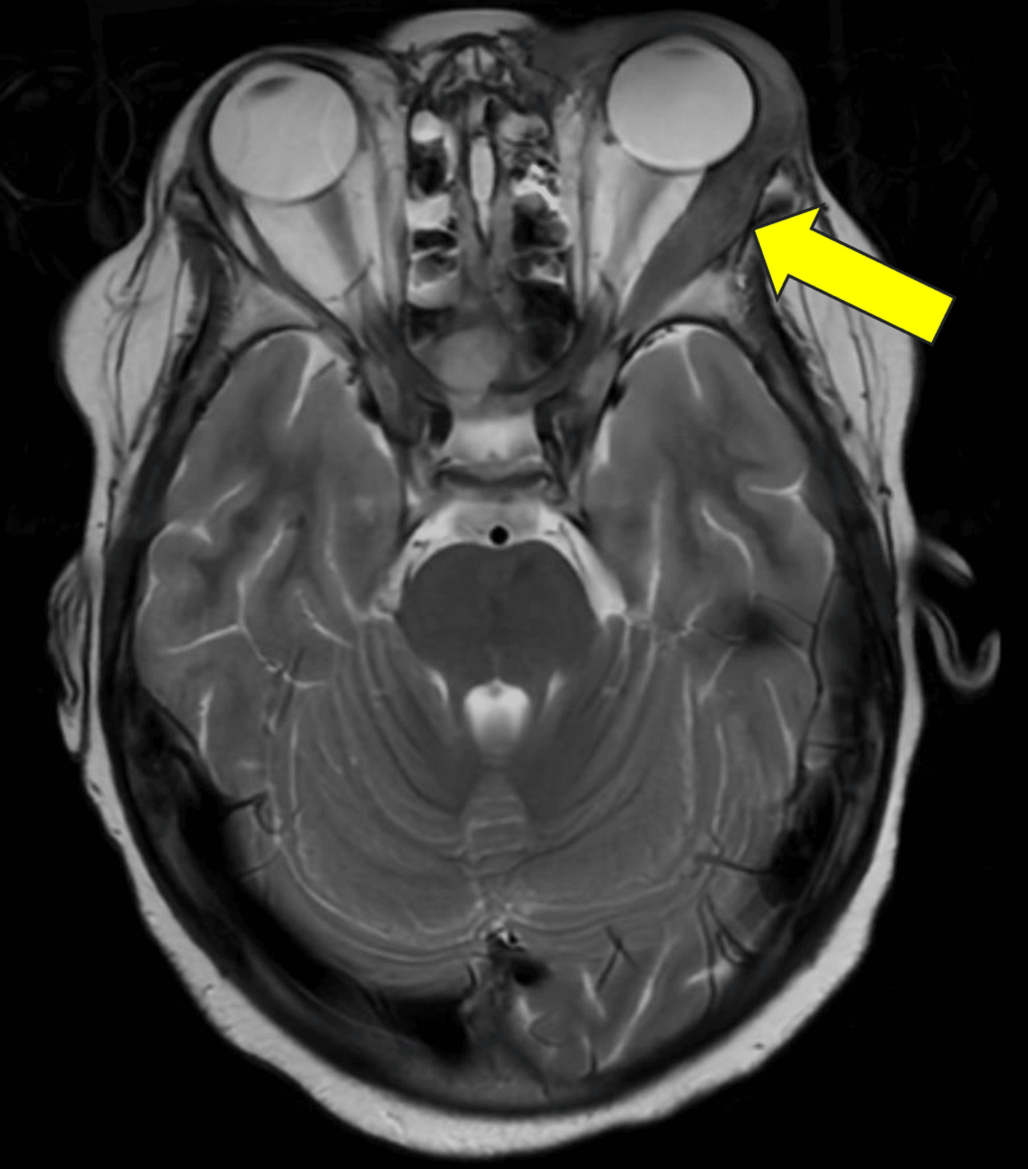
Axial T2-weighted MRI demonstrating a lesion involving the lateral rectus muscle and orbit of the left eye (yellow arrow).

Eighteen months after the completion of radiation to the left testicle, he presented with a left orbital mass, pain, and swelling with progressive proptosis. He underwent additional imaging studies, including a T2 MRI of the orbits, which revealed an orbital mass measuring approximately 1 × 5 cm and an intraorbital component along the lateral rectus muscle measuring approximately 4.1 × 0.9 × 2.1 cm, with an associated mass effect resulting in mild left proptosis (Figure 1).

**Figure 2:**
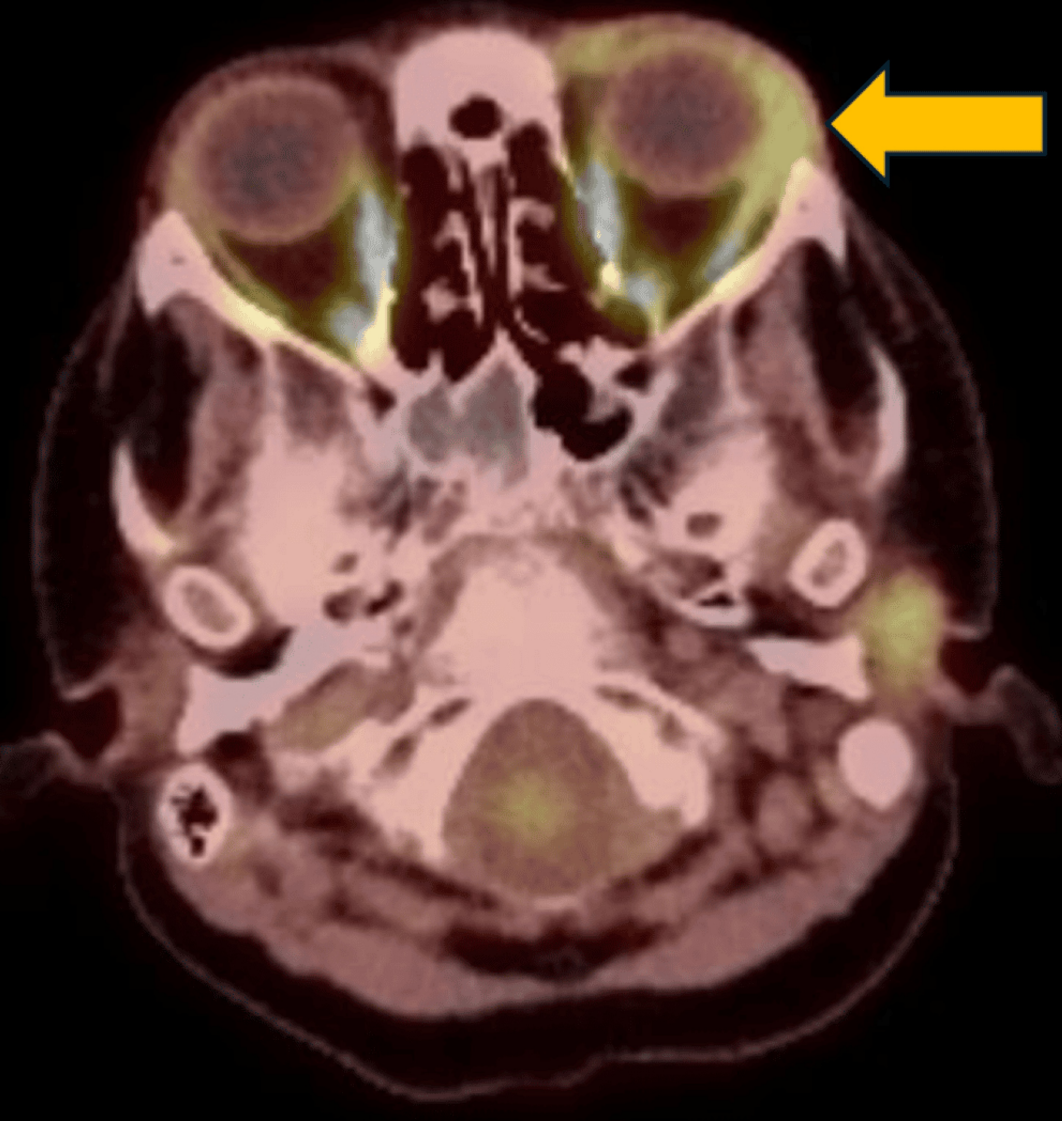
Pretreatment positron emission tomography scan showing a hypermetabolic lesion in the left orbit (yellow arrow), consistent with orbital myeloid sarcoma.

A biopsy of the orbital mass was consistent with MS. A positron emission tomography (PET) scan revealed increased uptake in the left lateral rectus muscle without evidence of metastatic disease (Figure 2).

Radiation planning and outcome

**Figure 3:**
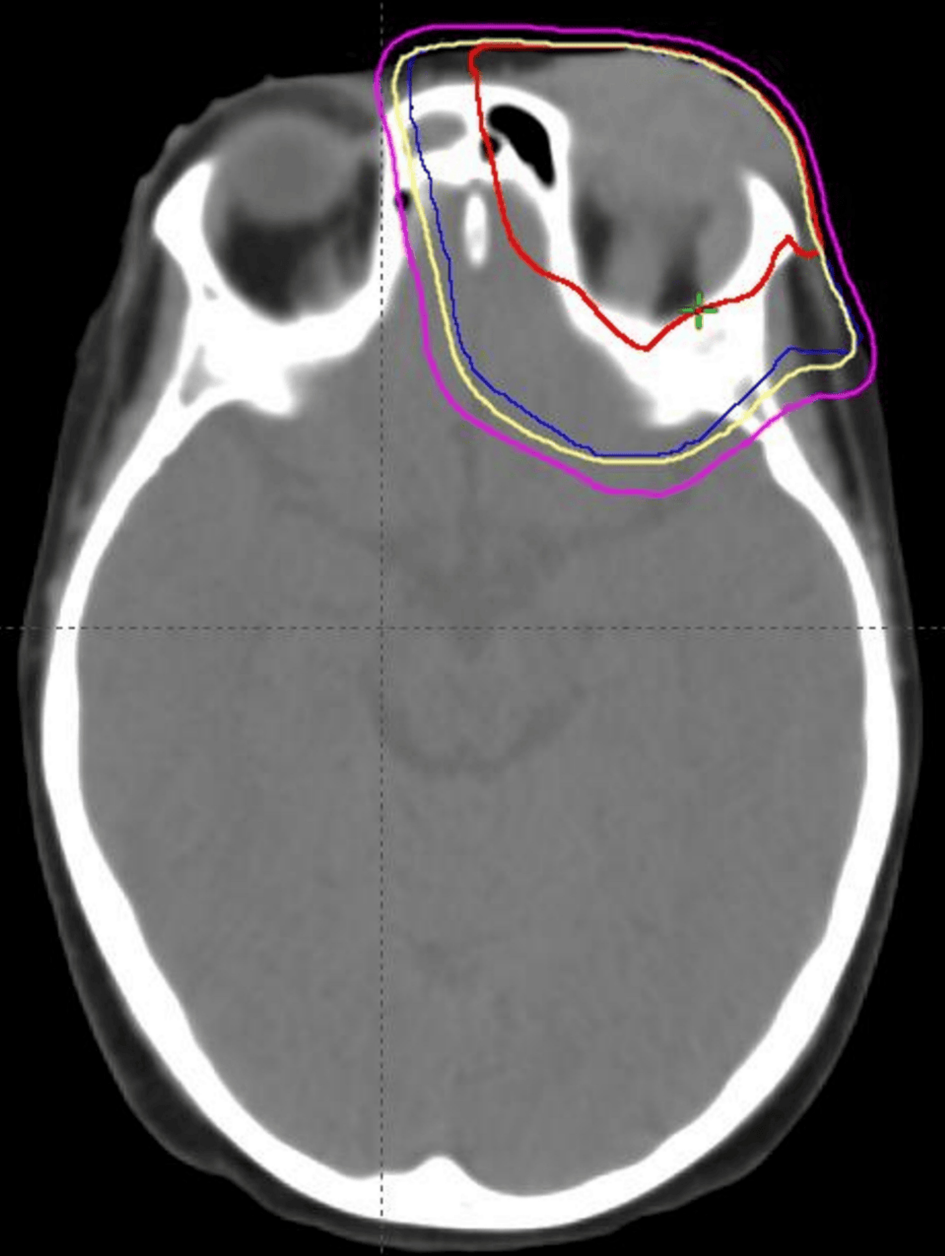
Radiation treatment planning image showing the gross tumor volume (red), planning target volume (blue), 95% isodose line (yellow), and 80% isodose line (magenta).

Local radiation therapy was planned, whereas systemic treatment options and a potential second SCT were considered. To address the orbital MS, the patient was treated with daily radiation therapy to the left orbit. Before the initiation of treatment, he underwent a planning CT scan without contrast, and an aquaplast mask was created to stabilize the head and neck. He underwent a CT scan using 3 mm slices. These images were downloaded to our treatment planning computer system so that a treatment plan could be developed. The gross tumor volume (GTV) consisted of the left orbit and any abnormality noted within the orbit, as well as the left lateral rectus muscle, as seen on the T2 axial MRI. The planning target volume (PTV) was a 1.5 cm margin around the GTV. The volumetric arc therapy was designed to treat the PTV while avoiding the right eye and orbit. He received 2,400 cGy in 12 fractions using volumetric modulated arc therapy (VMAT) to the left orbital lesion (Figure 3).

**Figure 4:**
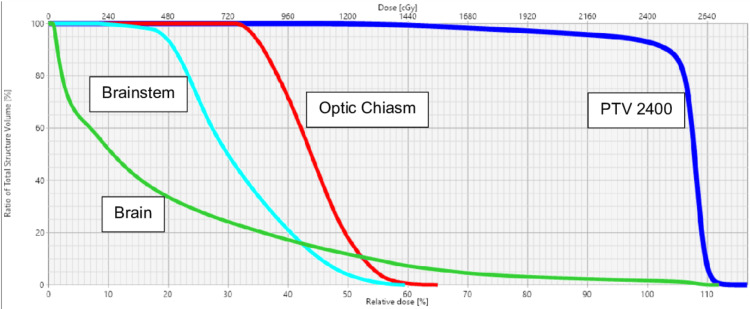
Dose-volume histogram illustrating the radiation dose distribution to the planning target volume (PTV, 2,400, dark blue), optic chiasm (red), brainstem (light blue), and brain (green).

The dose to the following structures (Figure 4) is displayed in the dose-volume histogram for the VMAT treatment using 6 MV photons.

The maximum dose to the brainstem was 1,432 cGy, the maximum dose to the optic chiasm was 1,561 cGy, and the maximum dose to the right and left cochlea was 525 cGy and 1,430 cGy. The mean dose to the PTV 2400 was 2,543 cGy.

The decision to treat the orbital MS with radiation therapy alone was based on several clinical considerations. Given his extensive prior treatments, including chemotherapy, HSCT, and ongoing management of chronic GVHD, his ability to tolerate additional systemic therapy was significantly compromised. Furthermore, this relapse was localized, with no evidence of systemic disease, as minimal residual disease (MRD) remained negative, and imaging showed no PET activity in prior sites. Radiation therapy alone was considered sufficient for local control while avoiding the additional toxicity and risks associated with concurrent chemotherapy in this vulnerable patient.

He did well post-completion of treatment, with a significant decrease in periorbital edema. He did have some hyperpigmentation of the skin and developed left eye conjunctivitis approximately one month post-treatment, which was treated with Polytrim and a three-day course of prednisone drops.

**Figure 5:**
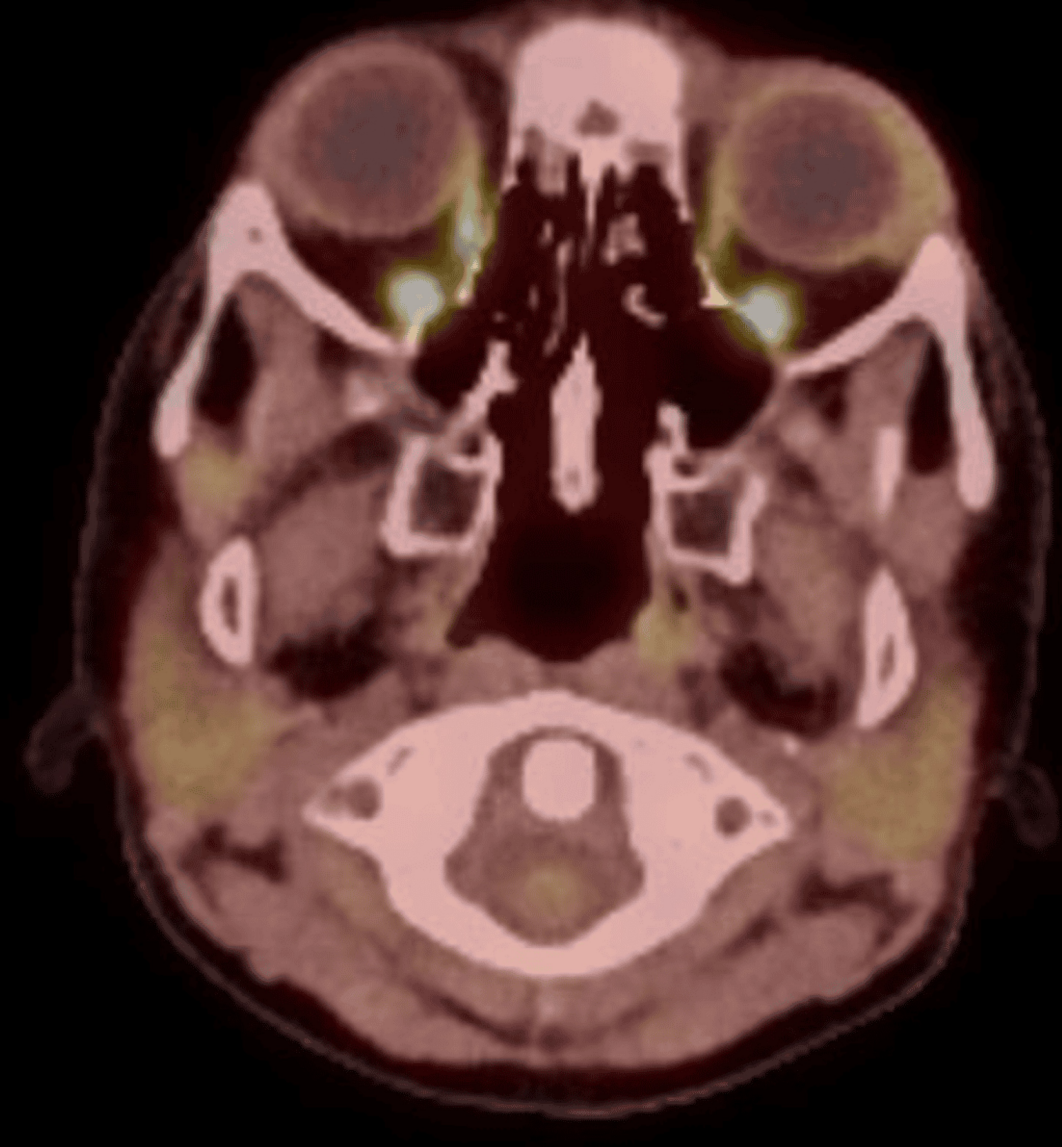
Post-treatment positron emission tomography scan showing near-complete resolution of the left orbital lesion, with normalization of fludeoxyglucose uptake in the affected area.

A PET scan just before the completion of treatment revealed resolution of the left orbital lesion, but interval appearance of numerous foci of focal fludeoxyglucose (FDG) activities in the osseous structures consistent with metastatic disease. He underwent venetoclax and azacitidine therapy to reduce tumor burden before the proposed transplant (Figure 5).

An orbital MRI obtained approximately six months after the completion of radiation therapy revealed complete resolution of the MS involving the left lateral rectus muscle and orbit. At a six-month follow-up visit, he had preserved visual acuity with no signs of optic neuropathy, retinopathy, or cataract formation.

## Discussion

This case highlights the challenges in treating relapsed extramedullary MS in pediatric patients following HSCT, particularly when systemic treatment options are limited. Although MS in the orbital region is rare, the skin and orbit are the most common locations for MS in pediatric cases [17]. Orbital MS presents unique therapeutic challenges due to the proximity of critical ocular and neurologic structures, requiring treatment that balances local control with preservation of vision and ocular function. As MS often signals an underlying or impending systemic relapse, systemic therapy is typically recommended even after the completion of AML treatment. Given the patient’s history of extensive systemic chemotherapy, HSCT, and chronic GVHD, treatment decisions required careful balancing of effective local control with minimizing systemic toxicity [13].

MS is known to be highly radiosensitive, making radiotherapy a vital treatment option. Local therapy primarily aims to deliver rapid symptom relief, which is observed in nearly 90% of patients with MS [12]. In this case, due to the patient’s history of intensive prior treatments, further systemic therapy was not feasible. The relapse was localized, with negative MRD and no FDG activity in previously involved sites. Therefore, radiotherapy alone was considered sufficient for local control while minimizing the risks and toxicity of concurrent chemotherapy.

Our patient received a total dose of 2,400 cGy, delivered in 12 fractions (200 cGy per fraction) over 18 days, using a 6 MV photon energy. The treatment plan adhered to conventional guidelines for radiation therapy, ensuring optimal coverage of the PTV while minimizing exposure to critical organs at risk, including the optic nerves, lenses, and brainstem. Dosimetric constraints were carefully met, with doses to the optic nerve (2,518 cGy), optic chiasm (1,213 cGy), and left lens (2,520 cGy) all within safe limits, minimizing late toxicity and preserving vision. By the end of treatment, the patient demonstrated a clinically complete response with no visual impairment. Long-term effects of orbital radiation therapy in children include cataracts, retinopathy, dry eye, and optic nerve damage. Literature supports that doses under 26 Gy with modern techniques (e.g., VMAT) reduce these risks [18-22]. However, continued ophthalmologic follow-up is necessary.

A potential consideration is whether a higher radiotherapy dose during the first relapse could have impacted the likelihood of later relapse and systemic progression. However, there is evidence that indicates no additional benefit in local control or systemic relapse prevention from escalating beyond 24 Gy, particularly in pediatric MS [6,12,14]. Therefore, dose escalation was not indicated, as higher doses could increase long-term toxicity without improving outcomes. In our case, despite complete local control of the orbital lesion following radiotherapy, the patient later developed systemic relapse involving distant osseous structures. This supports the concept that systemic relapses are driven more by disease biology and transplant history than by local radiation dose.

While this strategy was successful locally, its broader applicability is likely limited to patients with localized relapses and those who are contraindicated for systemic therapy. Alternative systemic options include venetoclax-based regimens, targeted FLT3 or IDH inhibitors, or a second HSCT where feasible [23-26]. Recent studies suggest that salvage regimens, such as FLAG-IDA plus venetoclax, hold promise in treating relapsed pediatric AML [27]. However, it has been demonstrated that radiotherapy does not improve long-term outcomes when combined with systemic therapy, as it has no significant impact on event-free or five-year survival rates [28,29].

Our patient was ultimately initiated on azacitidine and venetoclax for systemic relapse post-radiotherapy. Future studies should investigate the role of immunotherapies (e.g., anti-CD123, CAR-T) and maintenance therapy after HSCT in preventing recurrence. Multidisciplinary evaluation remains essential to tailor treatment to individual clinical scenarios.

Managing orbital MS in pediatric AML patients poses unique challenges, with varying treatment outcomes reported in the literature. Johnston et al. analyzed outcomes in pediatric AML patients with extramedullary leukemia, including orbital MS. They found that patients with orbital involvement had higher complete remission rates (96% vs. 78%) and overall survival (92% vs. 38%) compared to those with non-central nervous system extramedullary involvement, suggesting a more favorable prognosis for orbital MS [30].

In a separate case report, AlSemari et al. described two pediatric patients with isolated orbital MS without systemic AML, both of whom were managed with surgical orbitotomy and observation, resulting in no recurrence or progression [31]. This differs from our case, where the patient required radiotherapy following testicular relapse and was later diagnosed with orbital MS, reflecting a more aggressive disease trajectory and systemic relapse risk.

Johnston et al. also highlighted improved outcomes when combining systemic chemotherapy with local control measures [30]. However, systemic therapy was avoided in our patient due to chronic GVHD and poor treatment tolerance. Radiotherapy alone provided effective local control, highlighting its essential role when systemic treatment is contraindicated.

A retrospective study of 118 MS cases revealed that allogeneic HSCT was associated with significantly improved survival compared to non-transplanted patients. However, 36.4% of these patients experienced a relapse within a year [32]. Maintenance therapy with decitabine appeared beneficial after HSCT in some patients; however, this strategy was not pursued in our case due to the long-term immunosuppression requirements.

Maka et al.’s review of orbital MS supports a multimodal treatment approach for aggressive cases, typically involving the combination of radiotherapy with systemic chemotherapy [33]. However, they highlighted that the prognosis remains poor if systemic control is not achieved. Yet, when systemic disease is not present, radiotherapy alone can be justified. Our patient experienced a complete local response, although systemic relapse later emerged.

These cases collectively underscore the need for individualized treatment strategies tailored to disease location, systemic involvement, and patient-specific factors. Our case demonstrates the successful use of radiotherapy alone for local control, contrasting with the more aggressive combined therapies often used when systemic relapses are present. The dosimetry data reinforces the importance of precise radiation delivery to achieve effective local control while protecting adjacent critical structures.

## Conclusions

This case highlights the complexity of managing relapsed MS in AML patients post-HSCT. Treatment approaches must carefully balance achieving effective disease control while minimizing toxicity, especially in patients with a history of intensive therapy and side effects. In this case, radiation therapy alone (2,400 cGy in 12 fractions) was chosen due to the lack of systemic disease and the necessity to minimize additional systemic toxicity. The patient achieved a complete clinical response with no visual impairment, highlighting the role of targeted radiation therapy in localized MS relapses when systemic treatment is not feasible. This case supports the need for personalized therapy in relapsed extramedullary AML, showing the importance of precise dosimetric planning to improve local control while reducing long-term side effects. This approach may apply to other pediatric patients with localized MS relapse post-HSCT, particularly those with systemic toxicity concerns. Prospective studies are needed to evaluate radiotherapy-alone strategies in select cases and to assess emerging systemic options, such as targeted agents and immunotherapies.
